# Near-future ocean warming and acidification alter foraging behaviour, locomotion, and metabolic rate in a keystone marine mollusc

**DOI:** 10.1038/s41598-020-62304-4

**Published:** 2020-03-25

**Authors:** Rael Horwitz, Tommy Norin, Sue-Ann Watson, Jennifer C. A. Pistevos, Ricardo Beldade, Simon Hacquart, Jean-Pierre Gattuso, Riccardo Rodolfo-Metalpa, Jeremie Vidal-Dupiol, Shaun S. Killen, Suzanne C. Mills

**Affiliations:** 1 PSL Université Paris: EPHE-UPVD-CNRS, USR 3278 CRIOBE, BP 1013, 98729 Papetoai, Moorea French Polynesia; 2 Laboratoire d’Excellence “CORAIL”, Nouméa, Nouvelle-Calédonie, France; 3 University of Glasgow, Institute of Biodiversity, Animal Health and Comparative Medicine, Graham Kerr Building, Glasgow, G12 8QQ United Kingdom; 4 Australian Research Council Centre of Excellence for Coral Reef Studies, James Cook University, Townsville, Queensland 4811 Australia; 5 Pontificia Universidad Católica de Chile, Departamento de Ecología, Facultad de Ciencias Biológicas, Santiago, Chile; 6 Sorbonne Université, CNRS, Laboratoire d’Océanographie de Villefranche, 181 chemin du Lazaret, F-06230 Villefranche-sur-mer, France; 7 Institute for Sustainable Development and International Relations, Sciences Po, 27 rue Saint Guillaume, F-75007 Paris, France; 8 ENTROPIE IRD - Université de La Réunion - CNRS, Nouméa, 98848 Nouvelle-Calédonie France; 9 IFREMER, UMR 241 EIO, BP 7004, 98719 Taravao, Tahiti French Polynesia; 10 IHPE, Université Montpellier, CNRS, IFREMER, Université Perpignan Via Domitia, F-34095 Montpellier, France; 11 Present Address: Technical University of Denmark, DTU Aqua: National Institute of Aquatic Resources, 2800 Kgs, Lyngby, Denmark

**Keywords:** Non-model organisms, Behavioural ecology, Climate-change ecology, Ecosystem ecology, Feeding behaviour

## Abstract

Environmentally-induced changes in fitness are mediated by direct effects on physiology and behaviour, which are tightly linked. We investigated how predicted ocean warming (OW) and acidification (OA) affect key ecological behaviours (locomotion speed and foraging success) and metabolic rate of a keystone marine mollusc, the sea hare *Stylocheilus striatus*, a specialist grazer of the toxic cyanobacterium *Lyngbya majuscula*. We acclimated sea hares to OW and/or OA across three developmental stages (metamorphic, juvenile, and adult) or as adults only, and compare these to sea hares maintained under current-day conditions. Generally, locomotion speed and time to locate food were reduced ~1.5- to 2-fold when the stressors (OW or OA) were experienced in isolation, but reduced ~3-fold when combined. Decision-making was also severely altered, with correct foraging choice nearly 40% lower under combined stressors. Metabolic rate appeared to acclimate to the stressors in isolation, but was significantly elevated under combined stressors. Overall, sea hares that developed under OW and/or OA exhibited a less severe impact, indicating beneficial phenotypic plasticity. Reduced foraging success coupled with increased metabolic demands may impact fitness in this species and highlight potentially large ecological consequences under unabated OW and OA, namely in regulating toxic cyanobacteria blooms on coral reefs.

## Introduction

Rising atmospheric carbon dioxide (CO_2_) levels may lead to continued and accelerated global warming over the coming century^1^. The resulting elevated sea surface temperature (SST) (i.e. ocean warming; OW) is accompanied by increased partial pressure of CO_2_ (*p*CO_2_) in the ocean, thus rapidly changing the marine environment by increasing acidity (i.e. ocean acidification; OA) at unprecedented rates^2^. The Intergovernmental Panel on Climate Change (IPCC) has indicated that, by year 2100, global mean SST will increase by 2–4 °C and seawater pH decrease by 0.14–0.43 units^3^, with concomitant effects on marine ecosystems. Marine invertebrates are critical for ecosystem functioning^4^. Thus, any effects of OW and OA in the coming decades on the behaviour and physiology of marine invertebrates, particularly molluscs (see refs. ^5,6^), could substantially impact the biodiversity^7^ and functionality of marine communities and ecosystems, due to modifications in biotic interactions through changes in competition, predation, mutualism, or parasitism^8^, but few studies consider developmental acclimation of marine species to both OW and OA.

One way in which animals respond to a changing environment is through modifications to their behaviour^9^, and OW and OA affect a plethora of marine invertebrate behavioural traits^10^. Elevated *p*CO_2_ and/or temperature alter predator-prey behaviours in tropical^11,12^ and temperate^13–15^ gastropod and bivalve molluscs (e.g. ref. ^16^); activity, defensive^17^, and predatory^18^ behaviour in cephalopod molluscs; and foraging behaviour (e.g. ref. ^19^) and sound production^20^ in crustaceans.

Behavioural responses to a changing environment are also tightly linked with changes in physiology and organism metabolism^21^. Indeed, whole-animal metabolic rate is a key physiological trait with broad ecological relevance^22^ that is linked with reproductive performance, survival, and fitness^23^. Among other biotic and abiotic factors, elevated temperature and/or CO_2_ are well known to affect growth and metabolic rates in many marine species^24–26^.

Evidence is emerging, however, that some species can adjust to changing ocean conditions over time through phenotypic plasticity, or acclimation^27^. Acclimation may involve phenotypically plastic responses in behaviour, physiology, or morphology, which can help maintain fitness in a new environment^28,29^. An organism might maintain a specific trait (e.g. growth rate) in a sub-optimal environment compared with present-day conditions because of plasticity in underlying metabolic processes supporting that trait^30^. Acclimation can occur *via* reversible phenotypic plasticity within a given life stage, via developmental plasticity with persistent changes on adult traits, as well as through transgenerational plasticity reflecting the environment experienced by an animal’s predecessors^31^. Whereas reversible acclimation occurs over relatively short periods of several days to months, often within a life stage^32^, developmental acclimation occurs when exposure of an organism to a specific environment in its early life stages permanently modifies behaviour or physiology that enhances its performance in that environment later in life^33^.

Few studies consider developmental acclimation of marine species to both OW and OA^34^. To date, most studies on environmental change have focused on a single life stage of marine species placed more or less directly into future scenarios (i.e. reversible acclimation) (but see review in ref. ^35^). This “future shock” approach may shed light on species’ stress tolerance, but the observed sensitivity to stressors may be inaccurate^36^. Apart from these short-term approaches, experiments should ideally include multiple stages in the life cycle of a species (i.e. developmental acclimation)^37^, and even transgenerational acclimation^35,38^. As typical experimental setups are far from the slow progression of change in oceanic conditions, it is necessary to take these factors into account^39,40^. Whilst fish have been reported to show some developmental acclimation to warming and acidification^41^, no studies have investigated developmental acclimation responses to both these phenomena in molluscs.

**Figure 1:**
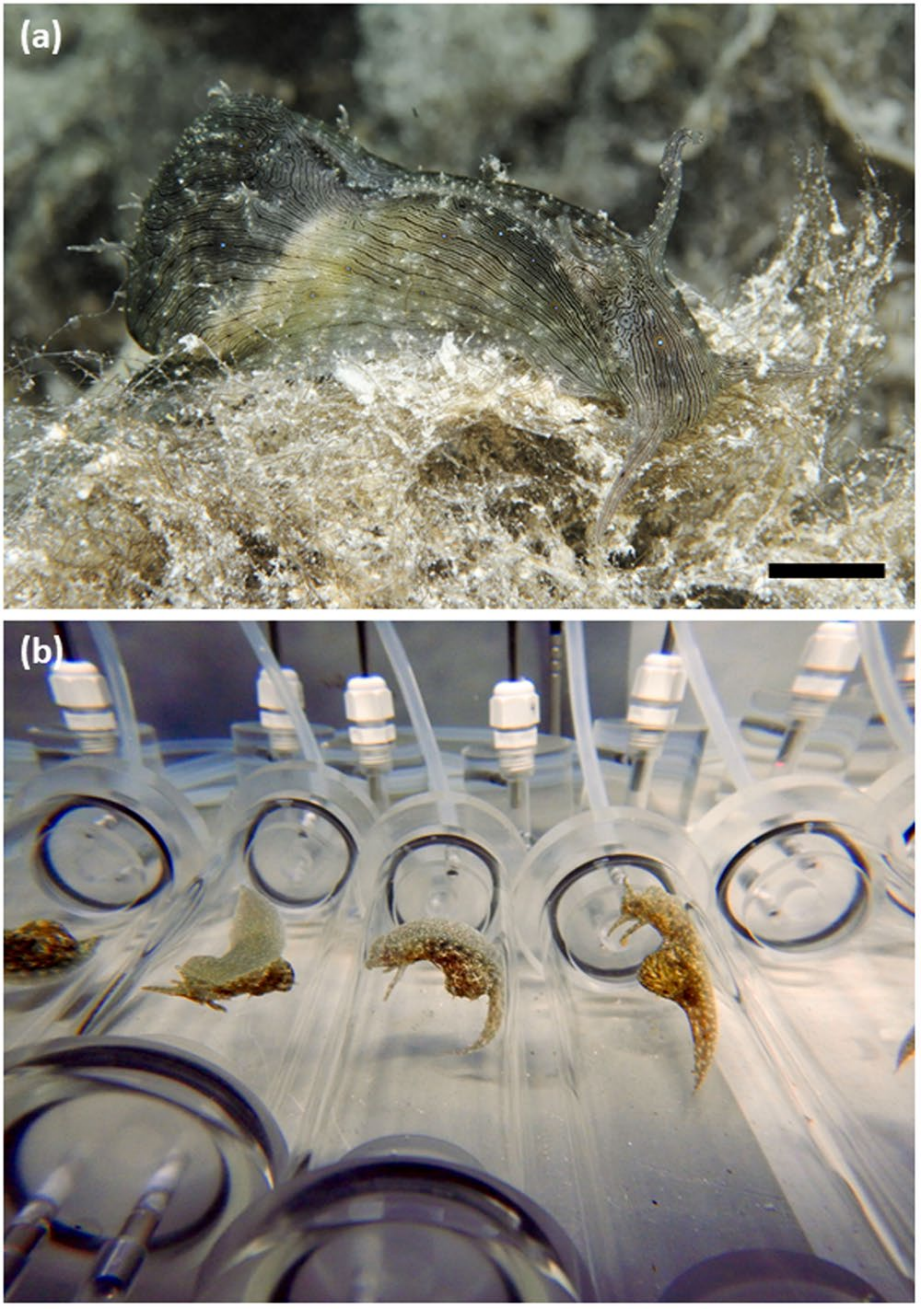
The circumtropical sea hare *Stylocheilus striatus*. (**a**) Photo of *S. striatus* foraging on the cyanobacteria *Lyngbya majuscula*. Photograph by Frederic Zuberer. Scale bar = 10 mm. (**b**) Photo of *S. striatus* in respirometry chambers. The optical oxygen probes placed in an external recirculation loop can be seen in the background.

Here, we investigate how projected near-future OW and OA affect locomotion and foraging behaviour, as well as metabolic rates, of a keystone marine mollusc, the circumtropical sea hare *Stylocheilus striatus*^42^ (Fig. 1). Specifically, we measure the behavioural and metabolic responses to a range of near-future warming and acidification conditions, both in isolation and in combination. We do this in adult sea hares that have been developmentally acclimated to four scenarios of OW and/or OA across three life stages (metamorphic, juvenile, and adult; see electronic supplementary material for an overview of *S. striatus* life stages) for a total of five weeks (developmental acclimation group), and we compare these to adult sea hares exposed to the same four scenarios of OW and/or OA for two weeks during their adult life stage only (adult acclimation group; representing a “future shock” experimental approach) or maintained under current-day conditions throughout their lives (control group). *S. striatus* was chosen for this study because it plays an important role in benthic coral reef ecology, particularly as the predominant grazer of the toxic cyanobacterium *Lyngbya majuscula*^43,44^ that has detrimental effects on human health including skin, eye, and respiratory irritation^45^, prevents the settlement of coral larvae^46^, and can cause phase shifts from coral to algal dominated reefs^47^. *S. striatus* also has a short post-settlement stage (~60 days) before reaching reproductive age and shows rapid growth (see Fig. S1 for duration times of the species’ developmental life stages). The difference of three weeks between our 5-week developmentally acclimated and 2-week adult acclimated sea hares not only spans different and multiple life-stages, but also represents at least a third of the post-settlement stage of these animals.

## Results

### Behavioural responses

#### Time to foraging choice

**Figure 2:**
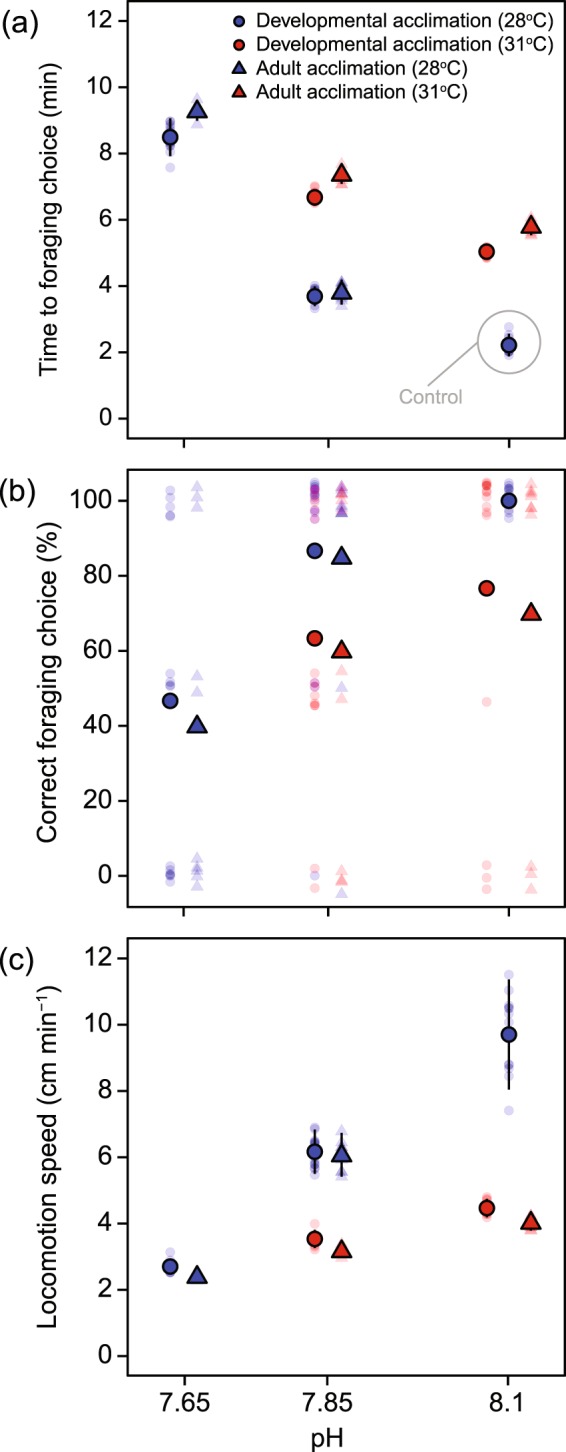
Behavioral responses of the developmental and adult acclimation groups of *Stylocheilus striatus* in T-maze trials. Mean and individual T-maze results showing (**a**) Time to foraging choice, (**b**) Correct foraging choice and (**c**) Locomotion speed of *S. striatus* from the developmental (circles; *n* = 15) and adult (triangles; *n* = 10) acclimation groups in each of the five treatments. Large symbols (circles or triangles) with black outlines are means ± SD and smaller, semi-transparent symbols are the mean of the two repeated measurements for each individual sea hare. Data points for the developmental and adult acclimation groups have been offset an even amount around each of their respective pH values (i.e. along the x-axis) to avoid overlap. Moreover, in panel (b), the data points for individual sea hares have values of 0% (incorrect choice in both technical repeats), 50% (one correct and one incorrect choice), or 100% (correct choice in both technical repeats) but have been offset along the y-axis for visual clarity.

Time to foraging choice of sea hares significantly increased with increasing severity of future climate change scenarios (Fig. 2a, Supplementary Table S3). For developmentally acclimated sea hares, the amount of time to reach and choose between the stimulus and control chambers increased from 2.22 ± 0.23 min for control animals to 3.69 ± 0.20 min in the low pH treatment (pH_NBS_ 7.85 and 28 °C) and finally to 8.49 ± 0.38 min for sea hares in the extreme pH treatment (pH_NBS_ 7.65 and 28 °C), showing a significant difference between all treatments (*p* < 0.0001 for all contrasts; Supplementary Table S3; Fig. 2a). The same pattern was observed for adult acclimated sea hares (*p* < 0.0001 for all contrasts; Supplementary Table S3; Fig. 2a).

Sea hares in the elevated temperature treatments took on average 2 mins longer to reach and choose between the stimulus and control chambers compared to sea hares in the control temperature, regardless of pH (Fig. 2a). The same pattern was observed for both acclimation groups, but adult acclimated sea hares always took on average 1 min longer to reach and choose between the choice chambers.

Comparison between the two acclimation groups showed that Time to foraging choice was significantly reduced for sea hares having undergone developmental acclimation compared to those that were only acclimated as adults (*p* < 0.0001), with the exception of animals subjected to the low pH treatment (pH_NBS_ 7.85 and 28 °C) (*p* = 0.52, Supplementary Table S5).

#### Correct foraging choice

Sea hares from all groups and treatments, except those that were developmentally acclimated in the extreme pH treatment (pH_NBS_ 7.65 and 28 °C) (*p* = 0.715), made an active choice between the stimulus and control cue chambers (all *p*-values <0.5; Supplementary Table S3). Furthermore, there was high repeatability of foraging choices between the two trials with the same animals (R = 0.711, p < 0.0001), indicating individual sea hare consistency in foraging choice, regardless of outcome (correct or not).

All sea hares in the control treatment chose the chambers with water that had been conditioned with cyanobacteria, i.e. in terms of foraging, 100% made the correct foraging choice (Fig. 2b). However, sea hares made more foraging choice errors as the severity of climate change conditions increased (Fig. 2b). For sea hares that experienced developmental acclimation, the percentage of correct choices was significantly lower in the combined (pH_NBS_ 7.85 and 31 °C), elevated temperature (pH_NBS_ 8.1 and 31 °C), and extreme pH (pH_NBS_ 7.65 and 28 °C) treatments when compared to control animals (*p* = 0.0094, *p* = 0.0487 and *p* = 0.0012, respectively). However, there were no significant differences when comparing the low pH (pH_NBS_ 7.85 and 28 °C) treatment with the control (see Fig. 2b). Similarly, the corresponding sea hares in the adult acclimated group also showed significantly lower foraging choice accuracy in the combined (pH_NBS_ 7.85 and 31 °C), elevated temperature (pH_NBS_ 8.1 and 31 °C) and extreme pH (pH_NBS_ 7.65 and 28 °C) treatments when compared to the control (*p* = 0.0079, *p* = 0.0255 and *p* = 0.0007, respectively), but not in the low pH (pH_NBS_ 7.85 and 28 °C) treatment (Fig. 2b; Supplementary Table S3).

Sea hares in the elevated temperature treatments (Fig. 2b) made on average 25% more foraging choice errors compared to sea hares in control temperatures, regardless of ocean acidification treatment (Fig. 2b). The same pattern was observed for both acclimation groups, with no statistically significant difference among developmentally and adult acclimated animals (*p* > 0.05 for all treatments, Supplementary Table S5).

#### Locomotion speed

Locomotion speed of control animals was 9.69 ± 1.46 cm min^−1^ (Fig. 2c). However, for developmentally acclimated animals, speed decreased with increasing severity of future climate change scenarios down to 2.69 ± 0.17 cm min^−1^ for sea hares from the extreme pH treatment (pH_NBS_ 7.65 and 28 °C) (*p* < 0.0001 for all contrasts; Fig. 2c; Supplementary Table S3). Similar results were found for adult acclimated sea hares, with speed decreasing from 6.06 ± 0.74 to 2.39 ± 0.07 cm min^−1^ from the low pH treatment (pH_NBS_ 7.85 and 28 °C) to the extreme pH treatment (pH_NBS_ 7.65 and 28 °C), respectively (*p* < 0.0001 for all contrasts; Fig. 2c; Supplementary Table S3).

Sea hares in the elevated temperature treatment (pH_NBS_ 8.1 and 31 °C) moved 6 cm min^−1^ slower compared to the control (pH_NBS_ 8.1 and 28 °C; *p* < 0.0001), but only 2.5 cm min^−1^ slower compared to animals in control temperature and low pH (i.e. pH_NBS_ 7.85 and 28 °C; *p* < 0.0001).

There were no significant differences in locomotion speed among developmentally and adult acclimated sea hares within the same OW and/or OA treatments (Fig. 2c; *p* = 0.102–0.981; Supplementary Table S5).

### Metabolic rate

**Figure 3:**
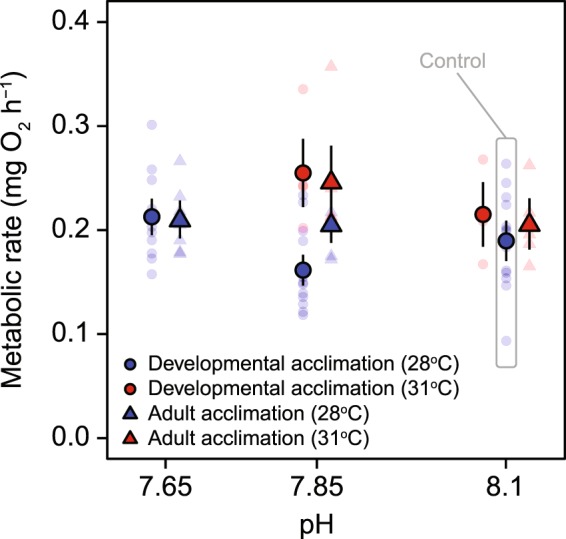
Body-mass-adjusted metabolic rate (oxygen consumption rate, ${\dot{M}}_{{O}_{2}}$) of the developmental and adult acclimation groups of *Stylocheilus striatus*. Large symbols (circles or triangles) with black outlines are means ± SD and smaller, semi-transparent symbols are values for individual sea hares. Sample sizes for each treatment are given in the main text. As for Fig. 2, data points for the developmental and adult acclimation groups have been offset an even amount around each of their respective pH values (i.e. along the x-axis) to avoid overlap.

The ${\dot{M}}_{{O}_{2}}$ of sea hares from reduced pH alone or elevated temperature alone scenarios were similar to those of control animals, at ~0.2 mg O_2_ h^−1^ (Fig. 3; Supplementary Table S3). However, the combined effect of low pH and elevated temperature (pH_NBS_ 7.85 and 31 °C) significantly elevated ${\dot{M}}_{{O}_{2}}$ of both developmentally acclimated (*p* = 0.0051) and adult acclimated (*p* = 0.0061) sea hares when compared to control animals, i.e. the combination of pH and temperature predicted for the year 2100 had a synergistic effect on sea hare ${\dot{M}}_{{O}_{2}}$ that was not observable for either stressor alone (Fig. 3). There were no significant differences in the ${\dot{M}}_{{O}_{2}}$ of sea hares between either the elevated temperature (pH_NBS_ 8.1 and 31 °C) or the extreme pH (pH_NBS_ 7.65 and 28 °C) treatments and the control for both developmentally and adult acclimated animals (Fig. 3; Supplementary Table S3), with the caveat that sample sizes were low for the elevated temperature groups (*n* = 4 for developmental acclimation and *n* = 5 for adult acclimation). Curiously, the treatment with low pH alone (pH_NBS_ 7.85 and 28 °C) tended to reduce the ${\dot{M}}_{{O}_{2}}$ of developmentally acclimated animals in comparison with the control, albeit this was not significant (*p* = 0.0624).

Nonetheless, the ${\dot{M}}_{{O}_{2}}$ from this low pH treatment (pH_NBS_ 7.85 and 28 °C) was significantly lower for developmentally compared to adult acclimated sea hares, while all other within-treatment comparisons between developmentally and adult acclimated groups showed no significant differences in ${\dot{M}}_{{O}_{2}}$ (*p* > 0.05; Supplementary Table S5; Fig. 3).

## Discussion

Our results show that behavioural traits linked to foraging and metabolic rates of a keystone marine mollusc are affected by exposure to future SST conditions^1^. Elevated CO_2_ (OA) and elevated temperature (OW) alone or in combination caused behavioural modifications, including increased time to foraging, incorrect foraging choices, and reduced locomotor speed. However, certain behavioural modifications were less severe for developmentally compared to adult acclimated sea hares, suggesting a potential for adaptive developmental plasticity to future climate change conditions that is not observed in animals subjected to short-term changes in their environment as adults. Furthermore, the metabolic rate of sea hares from both acclimation groups remained relatively unchanged to elevated CO_2_ or temperature alone, either due to a lack of physiological response or possible acclimation through plasticity, but in both cases the responses were similar during development as during adulthood. However, no acclimation in metabolic rate was found in both developmentally and adult acclimated sea hares when OA and OW were combined. Such increased metabolic rates, which are not compensated for with elevated foraging, place this opisthobranch mollusc at risk under future climate change conditions. As *Stylocheilus striatus* is the principal herbivore of toxic cyanobacteria in a trophic cascade on coral refs. ^43^, these effects can have considerable implications for ecosystem functioning, structure, and potentially human health.

Our results indicate that end-of-the-century projections of *p*CO_2_ and temperature may impair foraging by this important marine mollusc, decreasing locomotion speed and increasing time to foraging choice. Similar findings were described in the marine gastropod *Concholepas concholepas*, with increased *p*CO_2_ negatively affecting locomotor and sensory performance, thereby decreasing their foraging ability^48^. Generally speaking, high *p*CO_2_ levels appear to slow down the sea hares in our study, possibly due to the additive negative effects on cognitive, sensory (i.e. olfactory), and locomotor performance found in other organisms^49^. The combination of elevated temperature and elevated *p*CO_2_ has a synergistic detrimental effect on movement, as well as on decision making and time to foraging choice (Fig. 2), while increasing metabolic rate (Fig. 3). An increase in metabolic rates under combined elevated temperature and *p*CO_2_ conditions^50^, while overall foraging success declines, points towards energetic trade-offs (i.e. higher costs for the maintenance of cellular homeostasis and biochemical processes; ref. ^51^). It is possible that sea hares may move less due to a decreased energy surplus for movement and/or its altered cognitive and olfactory faculties to locate its food source. Additional work could determine the optimal movement speed of the sea hares (i.e. the speed at which they minimise their cost of transport) to evaluate the exact energetic trade-offs between movement costs and maintenance metabolism. Collectively, for all of the behaviours tested here, we found a greater effect under combined stressors as compared to individual stressors alone (Fig. 2). This is in line with previous works on the effect of multiple stressors on the behaviour of molluscs^48^ and fish^52^.

In addition to increasing foraging time, elevated *p*CO_2_ levels and/or temperature conditions also compromised decision-making when attempting to find food. The percentage of correct choices made by sea hares at elevated *p*CO_2_ levels and/or temperature conditions was 36 and 40% lower in the developmentally and adult acclimated groups, respectively, compared to the control (Fig. 2b). Decision-making was most disrupted in sea hares reared in extreme pH conditions (with correct choice being 53 and 55% lower, respectively compared to control animals). This behavioural effect may be related to a reduction in chemosensory ability, as reported in previous studies where elevated *p*CO_2_ levels was found to interfere with neurotransmitter receptor function, potentially GABA_A_-like receptors, thus compromising behaviour of snails^11,53^ and marine fishes^54^. Furthermore, in gastropods, a wide range of behaviours, including foraging, are mediated by chemical compounds known as secondary metabolites, and *S. striatus* are known to use the secondary metabolites produced by *Lyngbya majuscula* in foraging^55^. As such, disruption to decision-making may have arisen either from the compromised detection of stimulus cues (i.e. the perception of chemical cues^54,56^) or from an alteration of the chemical cues themselves^57^. Regardless of the mechanism, our results suggest that OA and OW disrupt the ability of this mollusc to identify its food source, thus potentially reducing food consumption.

Elevated *p*CO_2_, according to OA scenarios for the year 2100 and beyond, had no significant impact on metabolic rate of *S. striatus* at ambient temperature (28 °C). Similar results were found for another mollusc, the tropical humpbacked conch (*Gibberulus gibberulus gibbosus*)^58^, where there was no effect of elevated *p*CO_2_ on respiratory performance at any of the tested temperatures (range 28–33 °C) (also see ref. ^11^). On the other hand, elevated temperature resulted in a slight increase in metabolic rate of sea hares from both acclimation groups in the present study, however these were not significant. A similar case was found in another mollusc from the same region, the pearl oyster *Pinctada margaritifera*, where oxygen consumption was maximised at 30 °C but fell dramatically at 34 °C^24^. An increase in environmental temperature typically results in elevated metabolic rate in ectotherms, reflecting rate-enhancing effects of temperature on biochemical reactions^59^. However, temperature-dependence of metabolism is subject to acclimatory or evolutionary changes and thus also depends on the physiological state of the animals, as well as their environmental conditions and history of thermal adaptation or acclimation^59–61^. In the present study, a 3 °C increase in temperature (from 28 to 31 °C) under the current ambient CO_2_ conditions had no significant effect on the metabolic rate for either developmentally or adult acclimated *S. striatus*. This may reflect metabolic adaptation to a fluctuating temperature regime in intertidal and shallow water habitats as previously found in other molluscs^62^, and similar fluctuating conditions are found in Mo’orea (temperatures recorded from 2007–2016: min = 25.1 °C in October 2015; max = 30.8 °C in April 2016; see ref. ^63^).

The metabolic rate of sea hares exposed to OW and OA in combination was significantly higher than that of the control animals for both developmentally and adult acclimated animals, indicating that hypercapnia resulted in an elevated energy demand when combined with temperature stress. It is unlikely that the elevated metabolic rates were due to greater sea hare movement in the respirometry chambers, as sea hares showed reduced locomotion in T-maze trials with increasing severity of future climate change scenarios. Instead, evidence that the combination of OW and OA leads to elevated metabolic costs have also been shown in crustaceans^50^ and bivalves^64^, and is likely due to elevated physiological costs of the two stressors combined^65^. Importantly, if the energy demand of the sea hares is increasing while their foraging ability is decreasing, this may threaten future population sustainability and, consequently, ecosystem function.

Developmental plasticity is widespread among animals^66^, and several studies have demonstrated the importance of exposure to elevated temperature and/or *p*CO_2_ during early-life stages for the performance of adults^33,41^. Interestingly, in our study, when the behavioural and metabolic traits were compared between the developmentally and adult acclimated sea hares, both groups exhibited similar responses under OW and OA in almost all cases. However, the time taken to make a decision when identifying their food source was significantly shorter for developmentally acclimated individuals in all treatments, except for the low pH treatment (i.e. pH_NBS_ 7.85 and 28 °C; cf. Fig. 2a). Similar results were observed for correct foraging choice, although these were not significantly different (Fig. 2b). These results highlight the need to avoid “future shock” approaches in studies on climate change stressors that likely overestimate the sensitivity of species to stressors^36,67^. Even though this phenotypically plastic response during development may help *S. striatus* cope with foraging in future warmer and more acidic oceans, the negative effects of elevated *p*CO_2_ and/or temperature on the majority of the traits measured in this study were not alleviated following developmental acclimation. Emerging evidence demonstrates how plasticity of individuals can also depend on the environmental conditions experienced by previous generations^40^. When individuals are exposed to OW and/or OA, transgenerational acclimation has been reported to have positive effects on the performance of their offspring that experience the same conditions (e.g. refs. ^35,68^). Although such studies are laborious, future work on *S. striatus* should incorporate this aspect of acclimation if we are to truly understand the scope for phenotypic plasticity to assist this species to persist in the face of environmental change.

Overall, our findings indicate that elevated temperature and *p*CO_2_ conditions predicted for the future could reduce *S. striatus* foraging success by: (1) decreasing locomotion speed and movement, consequently increasing the amount of time taken to locate their food; (2) reducing the ability to detect a food source; (3) impairing decision-making; or (4) a combination of the modified foraging behaviours mentioned above. *S. striatus* is the primary herbivore in a coral reef trophic cascade, of which the cyanobacterium *L. majuscula* is the primary producer^44^. *L. majuscula* is known to harbour a potentially toxinogenic complex and produces more than 70 bioactive compounds, of which some are highly toxic^69^, causing multiple problems for humans including dermatosis, chelonitoxism, and food poisoning via the consumption of “infected” fish and turtles^45^. Furthermore, the cyanobacterium prevents the settlement of coral larvae^41^ and can cause phase shifts from coral to algal dominated refs. ^47^, hence any changes in the foraging behaviour of *S. striatus*, the principal herbivore in this trophic web, may have cascading impacts on the ecosystem. Combinations of behavioural abnormalities in keystone species such as this are likely to alter complex trophic interactions in marine food webs, which often involve multiple species and indirect effects^12,13,70^. Behavioural alterations, including modified foraging ensuing elevated *p*CO_2_ and temperature levels caused by global change, are likely to alter the fragile equilibrium of this food web structure with consequences that are difficult to predict.

The significant changes demonstrated here in behavioural and physiological traits of this marine mollusc under predicted near-future OW and OA may have significant ecological ramifications. As metabolic rates increase under warming and acidifying conditions (Fig. 3), sea hares may need to consume food of higher energetic/nutritional value or, alternatively, forage more in order to maintain their performance as environmental conditions change^65^. However, with impaired foraging skills as observed here, sea hares may be at risk. Individual performance may be directly affected *via* a shift in the balance of energy intake (reduced from lower foraging success) *vs*. expenditure (from higher metabolic rate), potentially impacting optimal foraging strategies^71^. Since *S. striatus* rely heavily on sensory functions for their survival and growth, compromised chemosensory function as a result of elevated *p*CO_2_ and temperature may affect the species significantly. One possible outcome may be a shift in species dominance from ‘sensory specialists’ (e.g. *S. striatus*) to more ‘sensory generalists’, which generally possess highly plastic behaviours and include detrimental invasive species^72^. Furthermore, elevated *p*CO_2_ and/or temperature may alter trophic interactions by reducing cyanobacterial palatability (as observed for macroalgae^73^ and seagrass^74^) and, consequently, cyanobacteria removal by *S. striatus* (*via* a reduction in foraging), although this needs to be tested.

Ultimately, a reduction in foraging as a result of impaired food cue detection under climate change, along with a potential reduction in food palatability, may affect the important role of sea hares for regulating toxic blooms of cyanobacteria on tropical coral reefs.

## Methods

### Sample collection and experimental treatment groups

In November 2016, 360 specimens of metamorphic *Stylocheilus striatus* (~3 mm in length; see electronic supplementary material for an overview of *S. striatus* life stages) were collected from Mo’orea lagoon (French Polynesia; 149°50′W, 17°30′S), at 1–2 m depths on sand flats within blooms of the cyanobacteria *Lyngbya majuscula* (Fig. 1a). Thirty sea hares were randomly assigned to each of twelve aquaria (40 L) at the CRIOBE research station, supplied with aerated running seawater (0.5 L min^−1^) at ambient pH and temperature (see electronic supplementary material for details on the flow-through seawater system) and were fed every two days with *L. majuscula* [3 g (wet weight) per sea hare] for the entire duration of the experiment.

After an initial laboratory habituation period (3 d) (Fig. S1), the temperature and/or pH of the seawater supplying the twelve aquaria were gradually changed (+1 °C day^−1^ and/or −0.1 pH unit day^−1^, respectively) over three days to obtain the five different treatment groups: (i) control (present-day pH and temperature; pH_NBS_ range 8.10–8.15 and 28 °C); (ii) low pH (pH_NBS_ target value 7.85 and 28 °C); (iii) elevated temperature (pH_NBS_ range 8.10–8.15 and 31 °C); (iv) low pH and elevated temperature (pH_NBS_ target value 7.85 and 31 °C); and (v) extreme pH (pH_NBS_ target value 7.65 and 28 °C). Out of the twelve aquaria, both the control (pH_NBS_ 8.10–8.15 and 28 °C) and low pH (pH_NBS_ 7.85 and 28 °C) treatments had three replicate aquaria, whereas the remainder of the treatments only had two replicate aquaria each due to laboratory space constraints. Low pH and elevated temperature were in accordance with the IPCC “business-as-usual” scenario (RCP 8.5) for 2100^1^. The pH of 7.65 was included as a more extreme OA scenario. Temperature, salinity, and carbonate chemistry data from experimental aquaria are summarised in Supplementary Table S1.

The sea hares were initially maintained in each of their respective treatments for three weeks, during which time they first transitioned from metamorphic into juveniles and finally into their adult stage (Fig. S1). After these first three weeks (i.e. upon reaching adulthood), 60 sea hares were randomly selected from the control treatment (thus leaving 30 control animals) and gradually acclimated (at the rates described above and in four temporary acclimation aquaria) in parallel with the developmentally acclimated animals to each of the four treatment conditions with modified temperature and/or pH to serve as the adult acclimation group (representing a “future shock” experimental approach). After reaching their target acclimation conditions (3 d), the 60 adult acclimated animals were removed from their temporary acclimation aquaria and placed in several labelled plastic tubs (fish breeding boxes; 20 ×10 ×10 cm) with large mesh panels to allow water circulation, which were floating in the replicate aquaria of the four treatment conditions with modified temperature and/or pH. All developmentally acclimated animals, including the remaining controls, were handled similarly; i.e. transferred from the main aquaria and placed in labelled plastic tubs of the same type floating in their aquaria. All animals were then kept in their respective seawater treatment for another two weeks (see Fig. S1 for experimental timeline), meaning that the developmentally acclimated animals experienced a total of five weeks acclimation, while the adult acclimated animals were only acclimated for two weeks. A period of two weeks was chosen to correspond to the average exposure time used in the majority of “future shock” experimental studies^67^.

### Carbonate chemistry

Temperature, pH_NBS_, and salinity in the aquaria were measured daily (YSI Professional Plus, Handheld Multiparameter Instrument) (Supplementary Table S1). Total alkalinity (TA) of seawater in the aquaria and mixing tanks was estimated by Gran titration (888 Titrando, Metrohm, Switzerland) from water samples taken weekly from each of the treatment tanks. All measurements and calculations are summarised in the electronic Supplementary Material Table S1.

### Behavioural responses

Behavioural experiments were performed in a T-maze setup to evaluate the locomotor speed and foraging responses of *S. striatus* to water-borne food stimuli (cyanobacteria in seawater) under each of the five treatments conditions (see electronic Supplementary Material Fig. S2 for details on the T-maze setup). At the start of each trial, each sea hare was habituated for 5 min by placing it at the base of the T-maze starting lane behind a grid preventing access to the rest of the lane. At the start of each trial, the grid was removed and we subsequently recorded (i) ‘Time to foraging choice’ (min), the time it took the sea hare to reach the end of the 15 cm long starting lane and first enter one of the T-maze arms (i.e. one of the choice chambers); (ii) ‘Correct foraging choice’ (Yes or No), whether the sea hare entered the choice chamber receiving seawater conditioned with *L. majuscula* or not; and (iii) ‘Locomotion speed’ (cm min^−1^), the time it took the sea hare to first reach a choice chamber regardless of which one it was.

Each trial lasted a maximum of 10 min, within which time all sea hares had made a choice between the stimulus or control cue chambers. The sea hare was then removed, water drained from the set-up, and the T-maze cleaned to avoid mucus trail following^48^. The T-maze setup was then refilled with water, but with the water sources switched between the arms, and the same sea hare was tested again in order to confirm that no directional bias could have affected the animals’ behaviour. Experiments were run for individual sea hares from both the developmentally and adult acclimated groups and at seawater pH and temperatures representative of their respective treatments.

### Metabolic rate

Metabolic rates of sea hares under each of the five treatments for both developmentally and adult acclimated groups were estimated from rates of oxygen uptake (${\dot{M}}_{{O}_{2}}$) using intermittent-closed respirometry [cf. ref. ^75^] (Fig. 1b, see electronic supplementary material for details). Respirometry experiments were performed in parallel with the behavioural trials and therefore on separate individuals. Unforeseen electrical problems resulted in sample sizes between 4–14 and 5–7 individuals per treatment for the developmentally and adult acclimated groups, respectively (Supplementary Table S2).

Sea hares were introduced into the respirometry chambers a few minutes before the first automated ${\dot{M}}_{{O}_{2}}$ recordings were started in the afternoon and remained there for 14.5‒16.7 h until the following morning, which produced between 72 and 83 ${\dot{M}}_{{O}_{2}}$ recordings per treatment. Prior to transferring sea hares to the respirometry setup, food had been withheld for ~20 h to ensure the sea hares were post-absorptive for the metabolic rate measurements. Upon completion of metabolic rate measurements in the morning, the sea hares were removed from their respirometry chambers, gently blotted dry, and weighed to the nearest mg (wet weight), and returned to their aquaria at their respective temperature and/or pH treatment.

### Data analysis and statistics

All statistical analyses were performed in R^76^. Differences between treatment groups in Time to foraging choice, Locomotion speed, and ${\dot{M}}_{{O}_{2}}$ were evaluated from linear mixed-effects (LME) models using the *lme4* package^77^, while differences between treatment groups in Correct foraging choice was evaluated from a generalised linear mixed-effects (GLME) model, also using *lme4*. Statistical significance (*p*-values) was evaluated from the *lmerTest* package^78^, which uses Satterthwaite approximations of degrees of freedom (further details, including model structures and outputs, can be found in the electronic supplementary material). In addition, chi-squared tests (two-sided) were performed to see whether choices (i.e. probability for success) from each treatment were or were not different from an equal expectation of an individual sea hare to enter either choice chamber of the T-maze (cf. Supplementary Table S4).

For comparisons of behaviours and metabolic rates between developmentally acclimated and adult acclimated groups, planned comparisons were carried out among each treatment pair using *glht* in the *multcomp* package and adjusted *p*-values are presented (cf. Supplementary Table S5).

Statistical significance was accepted at the *p* < 0.05 level. All the data are means ± SD.

## Supplementary information

Supplementary Information.
