# Supplementary material for: Near-future ocean warming and acidification alter foraging behaviour, locomotion, and metabolic rate in a keystone marine mollusc
Source: Sci Rep. 2020 Mar 25;10:5461. doi: 10.1038/s41598-020-62304-4 (PMC7096400; doi:10.1038/s41598-020-62304-4)
Supplement: Supplementary file 1 — Supplementary Information. [file 41598_2020_62304_MOESM1_ESM.docx]

**SUPPLEMENTARY MATERIAL**

**Near-future ocean warming and acidification alter foraging behaviour, locomotion, and metabolic rate in a keystone marine mollusc**

Rael Horwitz^1,2,γ^ *, Tommy Norin^3, γ^ **^,†^**, Sue-Ann Watson^4^, Jennifer C.A. Pistevos^1,2^, Ricardo Beldade^1,5^, Simon Hacquart^1^, Jean-Pierre Gattuso^6,7^, Riccardo Rodolfo-Metalpa^2,8^, Jeremie Vidal-Dupiol^2,9,10^, Shaun S. Killen^3^, Suzanne C. Mills^1,2^

***^†^****Present address: Technical University of Denmark, DTU Aqua: National Institute of Aquatic Resources, 2800 Kgs. Lyngby, Denmark*

*^1^ PSL Université Paris: EPHE-UPVD-CNRS, USR 3278 CRIOBE, BP 1013, 98729 Papetoai, Moorea, French Polynesia.*

*^2^* *Laboratoire d’Excellence “CORAIL”, Nouméa, Nouvelle-Calédonie, France*

*^3^ University of Glasgow, Institute of Biodiversity, Animal Health and Comparative Medicine, Graham Kerr Building, Glasgow, G12 8QQ, United Kingdom*

*^4^ Australian Research Council Centre of Excellence for Coral Reef Studies, James Cook University, Townsville, Queensland, 4811, Australia*

*^5^ Pontificia Universidad Católica de Chile, Departamento de Ecología, Facultad de Ciencias Biológicas, Santiago, Chile*

*^6^ Sorbonne Université, CNRS, Laboratoire d'Océanographie de Villefranche, 181 chemin du Lazaret, F-06230 Villefranche-sur-mer, France*

*^7^ Institute for Sustainable Development and International Relations, Sciences Po, 27 rue Saint Guillaume, F-75007 Paris, France*

*^8^ ENTROPIE IRD - Université de La Réunion - CNRS, Nouméa 98848, Nouvelle-Calédonie*

*^9^ IFREMER, UMR 241 EIO, BP 7004, 98719 Taravao, Tahiti, French Polynesia*

*^10^ IHPE, Université Montpellier, CNRS, IFREMER, Université Perpignan Via Domitia, F-34095 Montpellier, France*

γ The first two authors made an equal contribution to writing this paper.

*Corresponding author:

Rael Horwitz

Telephone: +972 54 6615787

Email: horwitzrael@gmail.com

***Stylocheilus striatus* biology**

*Stylocheilus striatus*, as common to most Aplysiidae, has five developmental stages (Fig. S1): (1) embryonic stage (fertilisation to egg hatching) lasting 5-7 days^1,2^; (2) planktonic stage (veligers feed on the phytoplankton) lasting a minimum of 30 days^2^; (3) metamorphic stage (larvae transform into benthic juveniles, which lose the velum and begin grazing on cyanobacteria using a radula) lasting 10-12 days, during which time the parapodia of *Stylocheilus* spp. grow over the shell and the shell is shed when the animal is ~8 mm in length^2^; (4) juvenile stage (after metamorphosis to reproductive maturity) lasting ~17 days^3^; and (5) adult stage. It is important to note that these estimates of developmental stage durations are based on observations of similar Aplysiid species and may differ due to species-specific traits and/or environmental conditions.

***Sample collection and experimental treatments***

The study was carried out in a seawater flow-through system, in which seawater pH was regulated using a pH controller (IKS Aquastar, Germany) connected to pH electrodes located in 300 L header tanks and calibrated on the National Bureau of Standards (NBS) scale. pH was manipulated by bubbling pure CO_2_ into seawater to reach the desired pH level. Temperature in the aquaria was controlled with V2 Therm 200 W digital aquarium heaters (Tropical Marine Centre). Small aquarium pumps (EHEIM compact 300, EHEIM GmbH & Co. KG, Deizisau, Germany) were used in each tank for water circulation.

***Behavioural responses setup***

The T-maze was made of PVC pipes (4 cm inner diameter) cut in half length-wise and consisted of a starting lane (the stem of the ‘T’; 15 cm in length) leading to two choice chambers perpendicular to the starting lane (the arms of the ‘T’; each 20 cm in length) that received incoming water at their external ends from two header tanks containing either seawater alone (control cue) or seawater conditioned with *L. majuscula* (stimulus cue). The flow rate into each arm of the T-maze was set to 100 mL min^−1^ using flowmeters. Cyanobacteria (200 g) was added to tanks containing seawater (10 L) 1 h prior to the experiment to condition the seawater. Sea hares were fasted 12 to 24 h before experiments.

This index of speed includes voluntary speed while in motion, but also includes time spent stationary, which was minimal in trials because all individuals were fasted and presumably motivated to feed.

***Metabolic rate setup and calculation***

The respirometry setup comprised a 40 L (water volume) tank receiving fully aerated flow-through seawater at the target pH and temperature from the 300 L conditioning tanks described above. There were eight 110 mL glass respirometry chambers in which the $\dot{M}_{O_{2}}$ of the sea hares was measured with the use of oxygen meters and probes (FireStingO2; PyroScience GmbH, Aachen, Germany), a peristaltic pump with gas-tight tubing that recirculated water through the chambers and past the oxygen probes, and a set of flush pumps that intermittently flushed fresh and fully aerated seawater through the respirometry chambers for 3 min in every 12 min intermittent-closed respirometry cycle (i.e. each closed measurement period lasted 9 min). Out of the eight respirometry chambers, at least one was always left empty to monitor background (microbial) respiration throughout each trial. The respirometry setup was shielded from surrounding disturbances by a large wooden board.

Respiration $(\dot{M}_{O_{2}}$ in mg O_2_ h^−1^) was calculated by first fitting a linear regression to the data for the decrease in oxygen concentration inside the respirometry chambers over 6.5 min during each of the 9 min closed phases of the respirometry cycles, after which the slopes from these regressions were multiplied by the volume of the respirometry chamber minus sea hare volume (determined by weighing the animals and assuming a density of 1 g mL^−1^). Since the sea hares did not exhibit elevated $\dot{M}_{O_{2}}$ after introduction to the respirometry chambers, as is generally seen for vertebrates (e.g. fish) due to the stress from being handled and moved (e.g. Fig. 2 in ref. 4 using the same setup), and also no pronounced flat-line indicative of a standard metabolic rate (e.g. ref. 5), the average (routine) metabolic rate of each individual sea hare was calculated as the mean $\dot{M}_{O_{2}}$ during the first six hours of each respirometry trial. The first six hours were chosen because background respiration became evident after this period.

***Data analysis and statistics***

Model structure was the same for each of the three behavioural traits, with either Time to foraging choice, Locomotion speed, or Correct foraging choice as the response variable, Treatment (with nine categories; one control and four temperature/pH treatments for each of the developmentally or adult acclimated groups), Length of sea hares, and Time of day as predictor variables (fixed effects), and Holding tank and Animal ID (since each sea hare was tested twice) as random effects. For Correct foraging choice, we ran a GLME on binomial data (logistic regression) but, since the sea hares always made the correct choice in the control treatment, we randomly assigned one observation as a wrong choice for the control since the logit function is undefined if the probability is exactly 1. Our analysis of these data is therefore a conservative estimate of the difference between the control treatment and the other treatments (but note that the observed value for the control treatment, i.e. 100% correct foraging choice, is presented graphically in the main article). For the $\dot{M}_{O_{2}}$ data, the LME had log_10_-transformed $\dot{M}_{O_{2}}$ as the response variable, log_10_-transformed Body mass and Treatment as the predictor variables, and Holding tank as a random effect. For all models, model selection proceeded by dropping variables one by one, starting with the variables with t-values closest to zero. Variables were kept in the models if their inclusion resulted in significantly better fit as indicated by log-likelihood ratio tests. The assumptions of homoscedasticity and normality of residuals were examined by visual inspection of residual-fit plots.

Repeatability (R) was calculated for the behavioural data (as each individual was tested twice) using the *rptR* package^6^. Values presented in the text of the main article are model estimates. For the behavioural data, length of sea hares was never significant in the models (*p* = 0.375–0.993) and these data are therefore presented graphically (cf. Fig. 3) as their raw (measured) values (i.e. no size-adjustments were performed). For the $\dot{M}_{O_{2}}$data, on the other hand, body mass was highly significant (*p* < 0.0001) and these data are presented graphically (cf. Fig. 3) as body-mass-adjusted values. Body-mass-adjustments of $\dot{M}_{O_{2}}$ of individual sea hares were achieved by adding the residuals from the linear regression of log_10_-transformed $\dot{M}_{O_{2}}$ *vs*. log_10_-transformed Body mass across all treatment groups to the $\dot{M}_{O_{2}}$ predicted from the regression for a 1 g (wet weight) sea hare. The developmentally and adult acclimated groups were combined in the regression since their scaling relationships were similar (developmentally acclimated: intercept = 0.200 mg O_2_ h^−1^, slope (scaling exponent) = 0.733; adult acclimated: intercept = 0.230 mg O_2_ h^−1^, slope = 0.733.

**Table S1: Temperature, salinity and carbonate chemistry of seawater.** Carbonate chemistry in present-day (pH 8.1) and acidified (pH 7.85 and 7.65) treatments was calculated from pH_NBS_, total alkalinity (TA), seawater temperature, and salinity using the program CO2SYS^7^, selecting the constants from ref. 8. Titration of TA standards were within 1% of that of certified reference material from Dr. A. Dickson (Batch No. 171; Scripps Institution of Oceanography). Mean concentrations, standard deviation (in parentheses) and number of replicates (in italics) are presented for each measurement. DIC = dissolved inorganic carbon, Ω_arag_ = aragonite saturation state.

| **Treatment** | **Temperature**  **(°C)** | **Salinity** | **pH_NBS_** | **TA**  **(µmol kg^−1^ SW)** | ***p*CO_2_**  **(µatm)** | **DIC**  **(µmol kg^−1^ SW)** | **HCO_3_^-^**  **(µmol kg^−1^ SW)** | **CO_3_^2-^**  **(µmol kg^−1^ SW)** | **CO_2(aq)_**  **(µmol kg^−1^ SW)** | **Ω_arag_** |
| --- | --- | --- | --- | --- | --- | --- | --- | --- | --- | --- |
| pH 8.1 + 28°C  (control) | 27.85  (0.1)  *70* | 36.29  (0.05)  *35* | 8.13  (0.02)  *70* | 2354.89 (14.48)  *12* | 466.3 (2.42)  *12* | 2044.55  (13.07)  *12* | 1813.21 (11.40)  *12* | 219.11 (1.53)  *12* | 12.22  (0.06)  *12* | 3.48 (0.86)  *12* |
| pH 7.85 + 28°C | 27.89  (0.15)  *70* | 36.28  (0.09)  *35* | 7.83 (0.03)  *70* | 2349.65 (24.4)  *12* | 1015.36 (10.68)  *12* | 2190.05  (23.32)  *12* | 2036.35 (21.65)  *12* | 127.1 (1.36)  *12* | 26.59  (0.27)  *12* | 2.02 (0.02)  *12* |
| pH 8.1 + 31°C | 30.91  (0.14)  *70* | 36.26  (0.11)  *35* | 8.12  (0.02)  *70* | 2349.17 (13.51)  *8* | 486.66 (3.8)  *8* | 2023.55  (11.65)  *8* | 1782.26 (10.1)  *8* | 229.4 (2.43)  *8* | 11.88  (0.1)  *8* | 3.71 (0.04)  *8* |
| pH 7.85 + 31°C | 30.88  (0.14)  *70* | 36.26  (0.06)  *35* | 7.86 (0.02)  *70* | 2355.61 (24.6)  *8* | 1026.63 (11.02)  *8* | 2178.1  (23.44)  *8* | 2014.25 (21.62)  *8* | 138.75 (1.48)  *8* | 25.09  (0.26)  *8* | 2.24 (0.02)  *8* |
| pH 7.65 + 28°C | 27.99  (0.1)  *70* | 36.305  (0.08)  *35* | 7.65 (0.03)  *70* | 2340.06 (7.56)  *8* | 1658.33 (5.48)  *8* | 2258.31  (7.43)  *8* | 2129.51  (7)  *8* | 85.47 (0.28)  *8* | 43.33  (0.14)  *8* | 1.36 (0.04)  *8* |

**Table S2:** Samples sizes (number of animals) for behavioural and metabolic rate measurements.

| Treatment | Developmental acclimation | Adult  acclimation |
| --- | --- | --- |
| **Behaviour** |  | |
| pH_NBS_ 8.1 + 28°C (control) | 15 | |
| pH_NBS_ 7.85 + 28°C | 15 | 10 |
| pH_NBS_ 8.1 + 31°C | 15 | 10 |
| pH_NBS_ 7.85 + 31°C | 15 | 10 |
| pH_NBS_ 7.65 + 28°C | 15 | 10 |
| **Metabolic rate** |  | |
| pH_NBS_ 8.1 + 28°C (control) | 13 | |
| pH_NBS_ 7.85 + 28°C | 14 | 6 |
| pH_NBS_ 8.1 + 31°C | 4 | 5 |
| pH_NBS_ 7.85 + 31°C | 5 | 6 |
| pH_NBS_ 7.65 + 28°C | 12 | 7 |

**Table S3:** Output of the final statistical models (i.e. excluding variables that did not significantly improve model fit) for the behavioural and physiological traits investigated. Estimates in parentheses are either back-transformed from logits to probabilities for correct foraging choice or from log_10_ to raw $\dot{M}_{O_{2}}$ values for metabolic rate. All pH target values for treatments are in the National Bureau of Standards (NBS) scale. [dev. acclim.] = developmental acclimation group, [adult acclim.] = adult acclimation group.

| **Response variable (bold) and fixed effects** | **Estimate** | **SE** | **df** | ***t* (LME)**  ***z* (GLME)** | ***p-value*** |
| --- | --- | --- | --- | --- | --- |
| **Time to foraging choice (LME model)** |  |  |  |  |  |
| (Intercept) pH 8.1 + 28^o^C [control] | 2.217 | 0.059 | 106 | 37.78 | < 0.0001 |
| pH 7.85 + 28^o^C [dev. acclim.] | 3.689 | 0.083 | 106 | 17.74 | < 0.0001 |
| pH 8.1 + 31^o^C [dev. acclim.] | 5.037 | 0.083 | 106 | 33.99 | < 0.0001 |
| pH 7.85 + 31^o^C [dev. acclim.] | 6.676 | 0.083 | 106 | 53.73 | < 0.0001 |
| pH 7.65 + 28^o^C [dev. acclim.] | 8.492 | 0.083 | 106 | 75.62 | < 0.0001 |
| pH 7.85 + 28^o^C [adult acclim.] | 3.817 | 0.093 | 106 | 17.25 | < 0.0001 |
| pH 8.1 + 31^o^C [adult acclim.] | 5.808 | 0.093 | 106 | 38.70 | < 0.0001 |
| pH 7.85 + 31^o^C [adult acclim.] | 7.378 | 0.093 | 106 | 55.63 | < 0.0001 |
| pH 7.65 + 28^o^C [adult acclim.] | 9.290 | 0.093 | 106 | 76.23 | < 0.0001 |
|  |  |  |  |  |  |
| **Correct foraging choice (GLME model)** |  |  |  |  |  |
| (Intercept) pH 8.1 + 28^o^C [control] | 3.367 (0.967) | 1.017 |  | 3.311 | 0.0009 |
| pH 7.85 + 28^o^C [dev. acclim.] | 1.872 (0.867) | 1.150 |  | ‒1.300 | 0.1935 |
| pH 8.1 + 31^o^C [dev. acclim.] | 1.189 (0.767) | 1.105 |  | ‒1.971 | 0.0487 |
| pH 7.85 + 31^o^C [dev. acclim.] | 0.546 (0.633) | 1.085 |  | ‒2.599 | 0.0094 |
| pH 7.65 + 28^o^C [dev. acclim.] | ‒0.134 (0.467) | 1.081 |  | ‒3.239 | 0.0012 |
| pH 7.85 + 28^o^C [adult acclim.] | 1.734 (0.850) | 1.194 |  | ‒1.367 | 0.1716 |
| pH 8.1 + 31^o^C [adult acclim.] | 0.847 (0.700) | 1.128 |  | ‒2.234 | 0.0255 |
| pH 7.85 + 31^o^C [adult acclim.] | 0.405 (0.600) | 1.115 |  | ‒2.657 | 0.0079 |
| pH 7.65 + 28^o^C [adult acclim.] | ‒0.406 (0.400) | 1.115 |  | ‒3.384 | 0.0007 |
|  |  |  |  |  |  |
| **Locomotion speed (LME model)** |  |  |  |  |  |
| (Intercept) pH 8.1 + 28^o^C [control] | 9.695 | 0.122 | 106 | 79.81 | < 0.0001 |
| pH 7.85 + 28^o^C [dev. acclim.] | 6.157 | 0.172 | 106 | ‒20.59 | < 0.0001 |
| pH 8.1 + 31^o^C [dev. acclim.] | 4.457 | 0.172 | 106 | ‒30.49 | < 0.0001 |
| pH 7.85 + 31^o^C [dev. acclim.] | 3.525 | 0.172 | 106 | ‒35.92 | < 0.0001 |
| pH 7.65 + 28^o^C [dev. acclim.] | 2.691 | 0.172 | 106 | ‒40.77 | < 0.0001 |
| pH 7.85 + 28^o^C [adult acclim.] | 6.065 | 0.192 | 106 | ‒18.90 | < 0.0001 |
| pH 8.1 + 31^o^C [adult acclim.] | 4.031 | 0.192 | 106 | ‒29.49 | < 0.0001 |
| pH 7.85 + 31^o^C [adult acclim.] | 3.173 | 0.192 | 106 | ‒33.96 | < 0.0001 |
| pH 7.65 + 28^o^C [adult acclim.] | 2.398 | 0.192 | 106 | ‒37.99 | < 0.0001 |
|  |  |  |  |  |  |
| **Metabolic rate (LME model)** |  |  |  |  |  |
| (Intercept) pH 8.1 + 28^o^C [control] | ‒0.711 (0.194) | 0.038 |  | ‒18.572 | < 0.0001 |
| Body mass | 0.730 | 0.109 |  | 6.708 | < 0.0001 |
| pH 7.85 + 28^o^C [dev. acclim.] | ‒0.777 (0.167) | 0.035 |  | ‒1.898 | 0.0624 |
| pH 8.1 + 31^o^C [dev. acclim.] | ‒0.666 (0.216) | 0.055 |  | 0.806 | 0.4235 |
| pH 7.85 + 31^o^C [dev. acclim.] | ‒0.573 (0.267) | 0.047 |  | 2.905 | 0.0051 |
| pH 7.65 + 28^o^C [dev. acclim.] | ‒0.657 (0.220) | 0.036 |  | 1.500 | 0.1387 |
| pH 7.85 + 28^o^C [adult acclim.] | ‒0.661 (0.218) | 0.045 |  | 1.125 | 0.2648 |
| pH 8.1 + 31^o^C [adult acclim.] | ‒0.670 (0.214) | 0.048 |  | 0.855 | 0.3958 |
| pH 7.85 + 31^o^C [adult acclim.] | ‒0.580 (0.263) | 0.046 |  | 2.841 | 0.0061 |
| pH 7.65 + 28^o^C [adult acclim.] | ‒0.641 (0.229) | 0.046 |  | 1.529 | 0.1313 |

**Table S4:** Results of the chi-squared tests (two-sided) for probability of success for sea hares making correct foraging choices. All pH target values for treatments are in the National Bureau of Standards (NBS) scale.

| **Treatment** | **Acclimation type** | **χ^2^** | **df** | ***p*-value** |
| --- | --- | --- | --- | --- |
| pH 8.1 + 28°C (control) |  | 30 | 1 | <0.0001 |
| pH 7.85 + 28°C | Developmental | 16.13 | 1 | <0.0001 |
| pH 7.85 + 28°C | Adult | 9.80 | 1 | 0.0017 |
| pH 8.1 + 31°C | Developmental | 8.53 | 1 | 0.0035 |
| pH 8.1 + 31°C | Adult | 3.20 | 1 | 0.0736 |
| pH 7.85 + 31°C | Developmental | 2.13 | 1 | 0.1441 |
| pH 7.85 + 31°C | Adult | 0.80 | 1 | 0.3711 |
| pH 7.65 + 28°C | Developmental | 0.13 | 1 | 0.7150 |
| pH 7.65 + 28°C | Adult | 0.80 | 1 | 0.3711 |

**Table S5:** Pairwise multiple comparisons of means between developmentally and adult acclimated sea hares within each treatment. All pH target values for treatments are in the National Bureau of Standards (NBS) scale.

| **Treatments compared** | **Estimate** | **SE** | ***t (metabolic rate)***  ***z (all other traits)*** | ***p-value*** |
| --- | --- | --- | --- | --- |
| **Time to foraging choice** |  |  |  |  |
| pH 7.85 + 28^o^C | ‒0.128 | 0.093 | ‒1.380 | 0.5200 |
| pH 8.1 + 31^o^C | ‒0.771 | 0.093 | ‒8.306 | < 0.0001 |
| pH 7.85 + 31^o^C | ‒0.702 | 0.093 | ‒7.566 | < 0.0001 |
| pH 7.65 + 28^o^C | ‒0.797 | 0.093 | ‒8.592 | < 0.0001 |
|  |  |  |  |  |
| **Correct foraging choice** |  |  |  |  |
| pH 7.85 + 28^o^C | 0.137 | 0.825 | 0.166 | 1.000 |
| pH 8.1 + 31^o^C | 0.342 | 0.652 | 0.525 | 0.974 |
| pH 7.85 + 31^o^C | 0.141 | 0.593 | 0.238 | 0.999 |
| pH 7.65 + 28^o^C | 0.272 | 0.585 | 0.465 | 0.984 |
|  |  |  |  |  |
| **Locomotion speed** |  |  |  |  |
| pH 7.85 + 28^o^C | 0.093 | 0.192 | 0.483 | 0.981 |
| pH 8.1 + 31^o^C | 0.426 | 0.192 | 2.220 | 0.102 |
| pH 7.85 + 31^o^C | 0.353 | 0.192 | 1.837 | 0.240 |
| pH 7.65 + 28^o^C | 0.293 | 0.192 | 1.527 | 0.418 |
|  |  |  |  |  |
| **Metabolic rate** |  |  |  |  |
| pH 7.85 + 28^o^C | ‒0.116 | 0.044 | ‒2.622 | 0.0428 |
| pH 8.1 + 31^o^C | 0.004 | 0.063 | 0.064 | 1.0000 |
| pH 7.85 + 31^o^C | 0.006 | 0.056 | 0.115 | 0.9999 |
| pH 7.65 + 28^o^C | ‒0.016 | 0.048 | ‒0.330 | 0.9953 |

**Figure legends**

**Figure S1: *Stylocheilus striatus* developmental life stages, photographs and experimental timeline.**

(**a**) The five developmental stages of *S. striatus* are shown: (1) embryonic stage (fertilisation to egg hatching); (2) planktonic stage (veligers feed on the phytoplankton); (3) metamorphic stage (larvae transform into benthic juveniles, which lose the velum and begin grazing on cyanobacteria using a radula); (4) juvenile stage (after metamorphosis to reproductive maturity); and (5) adult stage. The experimental timeline is also shown. Sea hares were exposed to their respective treatments for three weeks until reaching their adult stage (i.e. developmental acclimation; shown by diagonal grey stripes. Then, a set of adult sea hares reared in ambient conditions (control; pH 8.1 + 28°C) were transferred to each of the four treatments with modified temperature and/or pH to serve as the adult acclimated group. The two experimental sea hare groups were then kept in their respective seawater treatment for an additional two weeks (shown by diagonal grey stripes). A third group of individuals were maintained under control ambient conditions for the whole period. (**b**) Photographs of the five developmental stages of *S. striatus* are also provided.

**(a)**

**(b)**

**Figure S2: Diagram showing the T-maze setup.** The T-maze consisted of a starting lane (the stem of the ‘T’; 15 cm in length) leading to two choice chambers perpendicular to the starting lane (the arms of the ‘T’; each 20 cm in length) which received incoming water through a series of valves at their external ends from two header tanks containing either seawater alone (control cue) or seawater conditioned with the cyanobacterium *Lyngbya majuscula* (stimulus cue).

**
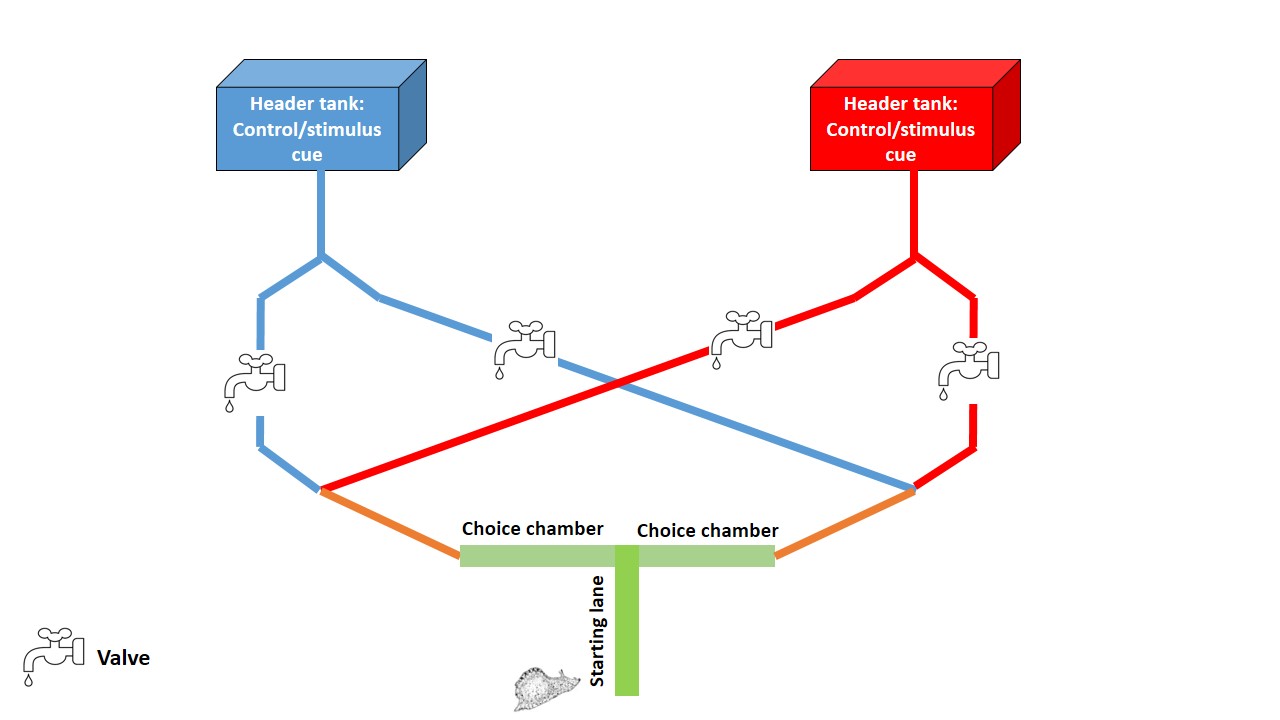
**

**References**

1. Horwitz, R., Jackson, M. D. & Mills, S. C. The embryonic life history of the tropical sea hare *Stylocheilus striatus* (Gastropoda: Opisthobranchia) under ambient and elevated ocean temperatures. *PeerJ* **5**, e2956 (2017).

2. Switzer-Dunlap, M. & Hadfield, M.G. Observations on development, larval growth and metamorphosis of four species of Aplysiidae (Gastropoda: Opisthobranchia) in laboratory culture. *Journal of Experimental Marine Biology and Ecology*, **29**, 245-261 (1977).

3. Kriegstein, A.R. Stages in the post‐hatching development of *Aplysia californica*. *Journal of Experimental Zoology*, **199**, 275-288 (1977).

4. Norin, T., *et al*. Anemone bleaching increases the metabolic demands of symbiont anemonefish. *Proc. R. Soc. Lond. B Biol. Sci.* **285**, 20180282 (2018).

5. Chabot, D., Steffensen,, J. F. & Farrell, A. P. The determination of standard metabolic rate in fishes. *J. Fish Biol.* **88**, 81-121 (2016).

6. Stoffel, M. A., Nakagawa, S. & Schielzeth, H. rptR: repeatability estimation and variance decomposition by generalized linear mixed-effects models. *Methods Ecol. Evol.* **8**, 1639-1644 (2017).

7. Pierrot, D. E., Lewis, E. & Wallace, D. W. R. MS Excel program developed for CO_2_ system calculations (Oak Ridge, TN: Carbon Dioxide Information Analysis Center, Oak Ridge National Laboratory, U.S. Department of Energy, 2006).

8. Mehrbach, C., Culberson, C. H., Hawley, J. E. & Pytkowicz, R. M. Measurement of the apparent dissociation constants of carbonic acid in seawater at atmospheric pressure. *Limnol. Oceanogr.* **18**, 897–907 (1973).
